# A qualitative study of attitudes towards, typologies, and drivers of concurrent partnerships among people of black Caribbean ethnicity in England and their implications for STI prevention

**DOI:** 10.1186/s12889-020-8168-0

**Published:** 2020-02-06

**Authors:** Sonali Wayal, Makeda Gerressu, Peter Weatherburn, Victoria Gilbart, Gwenda Hughes, Catherine H. Mercer

**Affiliations:** 1 Centre for Population Research in Sexual Health and HIV, Institute for Global Health, University College London (UCL), London, WC1E 6JB UK; 2 HIV & STI Department, Public Health England, Centre for Infectious Disease Surveillance and Control (CIDSC), Public Health England, London, NW9 5EQ UK; 3 The National Institute for Health Research Health Protection Research Unit (NIHR HPRU) in Blood Borne and Sexually Transmitted Infections at UCL in partnership with Public Health England (PHE) and in collaboration with the London School of Hygiene & Tropical Medicine, London, UK; 4 Sigma Research, Department of Social and Environmental Health Research, London School of Hygiene & Tropical Medicine, 15-17 Tavistock Place, London, WC1H 9SH UK

**Keywords:** Concurrency, Ethnicity, Sexually transmitted infection, Qualitative, Sexual behaviour

## Abstract

**Background:**

Partner concurrency, (having sexual partnerships overlapping in time), especially when condoms are not used, can facilitate sexually transmitted infections (STI) transmission. In Britain, STI diagnoses rates and the reporting of concurrency are higher among black Caribbeans than other ethnic groups. We explored attitudes towards, drivers, characteristics, and contexts of concurrent partnerships, and their implications for STI risk among black Caribbeans in England.

**Methods:**

Purposive sampling, by sex and age-groups, was used to recruit participants (overall *n* = 59) from five sexual health clinics and community settings in London and Birmingham, England. Audio-recorded four focus group discussions (*n* = 28 participants), and in-depth interviews (*n* = 31) were conducted (June 2014–December 2015). Transcribed data were thematically analysed using Framework Analysis.

**Results:**

‘Main plus’ and ‘non-main’ concurrency were identified in this population. Main plus concurrency involves an individual having a main partner with whom s/he has a “relationship” with, and the individual and/or their partner secretly or explicitly have other non-main partners. In contrast, non-main concurrency entails having multiple, non-committed partners overlapping in time, where concurrency is usually taken as a given, making disclosure to partners irrelevant. While main partnerships were usually long-term, non-main partnerships ranged in duration from a single event through to encounters lasting several months/years. Condomless sex was common with ex/long-term/married/cohabiting partners; whereas condoms were typically used with non-main partners. However, condom use declined with partnership duration and familiarity with partners. Awareness of partners’ concurrency facilitated condom use, STI-testing, and partner notification. While unresolved feelings, or sharing children with ex-partners, usually facilitated main plus concurrency; non-main concurrency was common among young, and single people. Gender norms, notions of masculinity, and sexual desires influenced concurrency. Black Caribbean popular music, social media, peer pressure, and relationship norms among black Caribbeans were also perceived to encourage concurrency, especially among men and young people.

**Conclusions:**

Concurrency among black Caribbeans is shaped by a complex interaction between emotional/psychological, interpersonal, sociocultural, and structural factors. Concurrency type, its duration, and awareness influence sexual health choices, and thus STI risk in this population. Collecting these data during clinic consultations could facilitate offering partner notification methods tailored to concurrency type. Gender- and age-specific, culturally-sensitive interventions addressing STI risks associated with concurrency are needed.

## Background

In Britain, since 2000 sexually transmitted infection (STI) surveillance and national probability survey data have shown that rates of bacterial STI diagnoses, for example: gonorrhoea, chlamydia, among people of black Caribbean ethnicity have remained higher than in people of other ethnicities [1–4]. Socio-economic deprivation and individual-level behavioural risk factors do not fully explain these ethnic inequalities in STIs [1, 4] suggesting the need to understand the role of sexual networks [4–6] in influencing STI risk. Sexual networks can influence whether STIs remain endemic within a population and explain inequalities in STI rates [5–7]. Specifically, patterns of partner concurrency, defined as overlapping sexual partnerships in which sexual intercourse with one partner occurs between two acts of intercourse with another partner [8], can influence not only the size but also the speed of spread of STIs in a population [9].

Research on HIV in the USA has shown that variations in the prevalence of concurrency and sexual mixing patterns by race exacerbated racial disparity in the epidemic potential [10]. Variation in the duration of concurrent partnerships also influences HIV transmission [11]. For example: long-term concurrent partnerships were associated with reduced HIV risk potentially due to the closed nature of the sexual networks [12]. Additionally, condom use in concurrent partnerships can influence STI transmission dynamics. Studies have shown that partnership type (i.e. steady or non-steady) can influence condom use [13, 14]. A high proportion of steady male partners of women diagnosed with *Trichomonias vaginalis* had concurrent partners and engaged in condomless sex with the women [15]. These data highlight the significance of understanding the nature and context of concurrent partnerships to better comprehend their implications for STI transmission [16].

In Britain, the prevalence of concurrency has been shown to be higher among black Caribbean than white British people [4]. Due to the dearth of data on concurrency in the context of STIs other than HIV generally and specifically among people of black Caribbean ethnicity, we conducted a qualitative study to explore the nature of concurrent partnerships among black Caribbeans in England. This study was undertaken as part of a larger study which sought to understand factors contributing to inequalities in sexual health as part of England’s National Institute for Health Research’s Health Protection Research Unit in Blood Borne and Sexually Transmitted Infections [17, 18].

## Methods

We conducted focus group discussions to explore attitudes towards and factors influencing concurrency among people of black Caribbean ethnicity, followed by one-to-one in-depth interviews to explore their personal experiences and their implications for sexual health choices and STI risk. People aged ≥15 years, who could read and speak English, and who identified as having a black Caribbean heritage were eligible to participate. The lower age restriction of 15 years was chosen because of the high prevalence of bacterial STIs among young black Caribbeans [19]. Due to variations in STI diagnoses rates by sex and age, participants were purposively sampled by sex and age groups (15–24, 25–34, ≥35 years). Participants aged < 16 years were only recruited from sexual health clinics because of the greater likelihood of them being sexually active, minimising ethical concerns related to discussing sexual behaviour.

Convenience sampling was used to recruit participants from five sexual health clinics and community settings such as colleges; with the help of community-based organisations working with black Caribbean communities on a range of issues such as depression, unemployment, teenage pregnancy, youth groups, and condom promotion. Based on analysis of England’s STI surveillance data, four sexual health clinics in London and one in Birmingham were chosen because a high proportion of attendees at these clinics are of black Caribbean ethnicity. Initial recruitment from community settings showed that most participants had no personal experiences of concurrency so subsequently in-depth interview participants were recruited only from sexual health clinics because clinic attendees are more likely to report STI risk behaviour, including concurrent partnerships than individuals in the general population [20].

Posters with contact details of the researchers were used to advertise the study in the sexual health clinics, community-based organisations and on Twitter so potential participants could directly contact the researcher. Black Caribbean community oriented commercial enterprises such as supermarkets, restaurants/take-away shops etc. were approached directly by researchers or targeted via Twitter (if they had a Twitter presence). Additionally, sexual health clinic and community-based organisation staff approached potential participants about the study, and passed their contact details to the research team. Subsequently a member of the research team contacted them to determine eligibility. Eligible participants were invited to participate in an in-depth interview or a focus group discussion in the city of their residence (no-one was recruited to participate in both), in a quiet place at either the participating sexual health clinic or the community-based organisation office. All participants were given a Study Information Sheet. Written informed consent was obtained. Parental consent of participants aged < 16 years was not sought due of the confidential nature of sexual health clinic services; however, participants < 18 years old were informed of our duties and legal limitations to confidentiality [21].

Piloted topic guides, informed by a systematic review [22], were used for the focus group discussions and interviews conducted during June 2014–December 2015. Female researchers with substantial qualitative research experience facilitated focus group discussions and conducted in-depth interviews. Each focus group discussion and in-depth interview lasted for approximately 60–90 and 50–60 min respectively and were audio-recorded. Participants were reimbursed for travel costs and received £20 as a token of appreciation.

Audio recordings were transcribed verbatim and thematically analysed using qualitative data analysis software NVivo 11 for Windows (QSR International Pty Ltd., Australia). We used Framework Analysis to inductively and thematically analyse data [23]. Firstly, two researchers familiarised themselves with a sub-set of transcripts and open-coded data using broad themes for key areas explored in the topic guides and developed sub-themes. These were revised based on discussion between the researchers and an index of themes was created which was applied to the entire dataset. Subsequently, all the data coded for a topic of interest, for example, “personal experience of concurrency” were retrieved and coded to refine sub-themes that summarised participants’ common and divergent views and experiences related to concurrency. Anonymised quotations are used to illustrate the analysis. We present quotes in the manuscript from a range of participants; however, sometimes quotes from the same participant are used more than once if their quote articulates the theme more clearly. We have also used terms and phrases quoted by individual participants where these express the sentiments of several participants for brevity so as not to disrupt the narrative’s flow. Our analysis draws on the ecosocial theoretical frameworks which purport that health inequalities arise from the interaction between individuals’ characteristics and their societal and ecological contexts which influence their behaviours [24, 25]. UCL Research Ethics Committee (Project ID: 6887/001) and National Research Ethics Service Committee of South Central-Oxford C (reference: 15/SC/0223) approved the study.

## Results

Four focus group discussions and 31 interviews were conducted from June 2014–December 2015 (Table 1). Altogether fifty-nine participants (*n* = 24 men) aged 15–70 years participated in our study. All the in-depth interview participants identified as black Caribbean whereas some focus group discussion participants identified as being from a black British or mixed ethnic background. None of the study participants identified as being gay or bisexual.

**Table 1:** Characteristics of study participants

*Focus group discussion participants characteristics*							
Number of focus group discussions	Men	Women	Total	Age group	Ethnicity	Area	Employment/occupation status
1	0	5	5	15–24	3 Black British and 2 mixed ethnicity (black Caribbean & English; black Caribbean & Turkish)	East London	All students
2	3	8	11	15–24	All, except 2 participants who identified as black Caribbean/black British, others identified as black Caribbean	North London	All students
3	2	3	5	35–48	3 participants identified as black British and 2 as black Caribbean	West London	2 were unemployed, 2 employed, 1 other (HIV+ and receiving benefits)
4	4	3	7	> 48	Except one participant who identified as black British, others identified as black Caribbean	South London	People living with mental health problems and were largely unemployed/voluntarily retired
Total	9	19	28	–	–	–	–
*In-depth interview participants characteristics*							
				*Men*		*Women*	*All*
Age group							
15–24				5		6	11
25–35				5		5	10
> 35				5		5	10
Recruitment site							
Sexual health clinics				10		12	22
Community settings				5		4	9
Location							
London				9		14	23
Birmingham				6		2	8
Total				15		16	31

### Typology and characteristics of concurrent partnerships

Overall, two partnership types ‘main/regular’ and ‘non-main/casual’ were identified from the focus group discussions and the personal experiences shared by the interview participants. A main/regular partner is typically someone with whom a person has *“a relationship”*, an “*emotional connection”* to. These feelings are mutual, and as such, there is an expectation of exclusivity of sexual relationship. In contrast, a non-main/casual partnership entails being with a person primarily for *“having sex”, “fun”*, without any commitment or expectation of exclusivity*.* Two key typologies of concurrency identified were ‘main plus’ and ‘non-main’ concurrent partnerships:

### Main plus concurrent partnerships

These were characterised by an individual having a main/regular sexual partner, and they and/or their partner having non-main/casual partner(s) concurrently. These partnerships could be: i) ‘closed from one-end’ where only one partner in the ‘main’ partnership has other non-main sex partner(s), which the main partners are either aware or unaware of, ii) ‘open-ended’ where both partners in a main partnership implicitly or explicitly agree to have sex with non-main partners, either together (i.e. threesomes/group sex) or individually. These partnerships were perceived to be more common among black Caribbean men than women. Women were more likely to be in ‘closed on their-end’ concurrent partnerships. Main plus partnerships were usually of longer duration or recurrent. Participants used terms such as *“side chick”, “thot”, “jez”* to refer to men’s non-main concurrent female partners*,* and *“side boy”, “side dick”* to refer to women’s non-main concurrent partners which highlights an awareness of existence of concurrency in this population:

*Participant 1: I suppose it does happen (having different sexual partners at the same time), because there’s the thing that they’ve got a side chick and all that; like if they’re in a relationship then they’ll have another girl and another girl and another girl.*

*Facilitator: Sorry, what did you mean by a side chick?*

*Participant 1: You have like a main chick, side chick, mistress …*

*Participant 2: yeah, you’ve got loads of faces. So if you’re in a relationship, you’ve got obviously the main girl that you’ve obviously got an emotional connection with, but then you’ve got something on the side, and then you’ve got something else on the side!*

*Participant 3: It’s not just with guys as well, it could be girls. This is another thing, you can’t say it’s just like a side girl, there are side boys, you know?*

*Participant 2: You get side men.*

***Focus group 1, young women aged 15-24 years, East London***

However, the majority of participants described main plus concurrency to be *“kind of wrong”*. Some women felt that if they were to find out about their main male partner’s concurrency it would *“make (you) think (you) are not good enough”, “make (you) question (your) womanhood”.* Similarly, some men felt that awareness of their main female partner’s concurrency would make them feel inadequate: *“(you’d) start feeling like a wimp or (you’re) not manly enough”*. Some men also felt that it could lead to violence with her concurrent partner, and potentially, relationship break-up.

### Non-main concurrent partnerships

These were characterised as having multiple non-main partners overlapping in time. Several focus group and interview participants perceived such partnerships to be common among young people and single people regardless of their age. However the importance of being honest about *“if people/they (are) in an open relationship”* with sexual partners and to give the other person the *“choice”* to decide if they want to be in this situation was emphasised, although they also recognised that this may not necessarily happen. In contrast, some interview participants felt that disclosure of their concurrent partnerships to non-main partners was irrelevant because they were *“not in a relationship”* with them nor answerable to them.

Sometimes participants assumed that their non-main partners would know that their sexual partnership was not exclusive. Some focus group and interview participants felt that if a person is not in a *“serious relationship”* then having partners concurrently would be acceptable because they would *“not be hurting anyone”*. However, mismatch of expectations about the nature of relationship between non-main partners, where one person may want to explore the potential of having a committed *“emotional relationship”,* whereas the other does not, was also highlighted*.* In terms of duration, such types of partnerships were long-term, involving regular or occasional meetings for sex, with participants describing these partners as “*fuck buddies” or “friends with benefits”*. In contrast, some were spontaneous, brief partnerships, involving one-off sex through to several weeks.

### Impact of concurrent partnership typologies on sexual health related behaviours

Concurrency typology and its characteristics influenced sexual health choices and STI risk in several ways.

#### Condom and contraception use

Some participants in main plus concurrent partnerships did not usually use condoms with their long-term main partner, despite being aware of their partner’s concurrency:

*Interviewer: What type of partnership did you have with him?*

*Participant: Always closed on my side. He was already with somebody else when I got with him, because when you’re young, you don’t care about stuff like that. So, yeah, he was obviously sleeping with somebody else and me at the same time. He was my first love. I really fell hard for him. I can honestly say I wasn’t thinking about it from a sexual point of view with regards to infection and diseases.*

*Interviewer: How did you know that he had another partner?*

*Participant: Because he did, that’s how I met him, he was a family friend, like he knew my dad.*

*Interviewer: So was he married or was he seeing somebody?*

*Participant: No, he wasn’t married, he was co-habiting.*

*Interviewer: Did any of your ex-partners’ (participant had concurrent partnerships of similar nature at different points in her life with different partners) other partner find out that they were seeing you?*

*Participant: Not to my knowledge.*

*Interviewer: And did you use condoms with him?*

*Participant: No because I was in love and I was stupid.*

***Interview 1, female aged >35 years, Birmingham***

Some participants felt that in main plus partnerships, the awareness of a partner’s concurrency could lead to women - usually as the non-main partner - trying to get pregnant to *“trap”* their partner, thus leading to not using contraception nor condoms.

In non-main partnerships, pregnancy prevention was usually not discussed; however, some participants felt that in non-main concurrent partnerships, the *“no strings attached”* nature of these partnerships facilitated condom use:

*Participant: In the summer time, I had one or two other partners.*

*Interviewer: OK. And how old were they?*

*Participant: One was 23/24 and one was 18/19.*

*Interviewer: And you were seeing both around the same time?*

*Participant: Yeah.*

*Interviewer: And they were both women?*

*Participant: Yeah.*

*Interviewer: And what kind of partnership did you have with them?*

*Participant: Sexual.*

*Interviewer: So you didn’t have any emotional relationship with them in a way?*

*Participant: Well, in a way, but not serious.*

*Interviewer: And how long did your relationship with them last?*

*Participant: About two months or three months; I’m not too sure to be honest.*

*Interviewer: And did you use condoms with these partners?*

*Participant: Yes.*

*Interviewer: Why did you use condoms with these partners?*

*Participant: Because they weren’t my girlfriend. Or I didn’t make them my girlfriend. But that’s what I normally do when I don’t have a girlfriend, so … yeah.*

***Interview 10, male age range 15-24 years, East London***

*Female participant 1: I think a relationship is like more you don’t use the condom or whatever, but friends with benefit, because you’re not actually going out with the person, you’re just sleeping with the person or whatever, you use it more or whatever.*

*Facilitator: What do others feel about what she just said?*

*Male participant 1: I’d do the same thing. If I was just sleeping with a person, then I would be more protective, but if I was in a relationship and then not*

*Female participant 3: I think because as well you don’t know if they’re sharing sex with other people (murmurs of agreement) because from the get-go you only know it’s not a thing like that …*

***Focus group 2, mixed sex participants’ age range 15-24 years, North London***

Some participants however also shared that the likelihood of condom use with non-main partners declined with increasing partnership duration and familiarity between partners. Condoms were usually used with one-off partners, but inconsistently in the event of alcohol consumption, and dependant on the degree of sexual attraction towards the person.

#### STI testing and partner notification

In main plus concurrent partnerships, awareness of partners’ concurrency - regardless of whether it was their main or non-main partner - usually led an individual to test for STIs, especially if they had previously been diagnosed with an STI due to a partner *“cheating”* on them:

*Interviewer: So why did you come to the clinic this time?*

*Participant: This time I came because my partner stepped out and he says that he used protection, but I don’t believe that he did, because I know him! And obviously we’ve been having sex unprotected (because partner does not like using condoms). So I’m just taking my precautions and going and checking it out just to make sure that everything is OK down there.*

*Interviewer: So how did you know he’d stepped out?*

*Participant: The girl messaged me and told me about it, so that’s how I found out that they saw each other and that they had sex. So it’s been a bit of a tough one but, again, you’ve got to put your feelings aside to a degree and just focus on getting things sorted out. So that’s where I’m at right now.*

***Interview 11, female age range 25-35 years, East London***

Whereas participants who were/had been in non-main concurrent partnerships said that they tested regularly, usually at the end of a sexual relationship and/or at the beginning of a new partnership, sometimes along with their new partner.

With regards to notifying the main partner following STI diagnosis, fear of losing them if they were unaware of an individual’s concurrent partners was a barrier. However, some participants had done so due to concerns of re-infection. Partner notification sometimes led to partners blaming each other for having concurrent partners. It also often led to them breaking-up, especially if the main partner was relatively new and there had been no prior awareness of the partner’s concurrency:

*Interviewer: Have you ever asked anybody to go to a clinic when you’ve found out that you’ve had an infection?*

*Participant: Yeah. Like every time I’ve ever had an infection, I’ve, like, just contact them, phone them up and say, “You know what, I’ve got something to tell you, basically I’ve got an infection and you need to go to the clinic.” Like before when it happened to me, like when that girl gave me something, I had a relationship and I split up through that. Like, she was like my ex-girlfriend and then obviously I slept with this regular girlfriend and then I had to tell my regular girlfriend that I caught something off that one, and then that split up my relationship.*

*Interviewer: How long had you been together in this regular relationship?*

*Participant: Not long, you know, about eight months.*

***Interview 7, male aged 25-35 years, Birmingham***

In some situations, developing symptoms had prompted individuals to test for STIs, and confront their partner about sexual concurrency in the event of STI diagnosis. A few participants also described being diagnosed with an STI after they had had sex with a main partner who lives abroad and/or new non-main partners they met whilst abroad, mainly in the Caribbean. They had usually informed the main partner of the STI diagnosis; however notifying non-main partners was challenging due to limited, if any, contact details. Unwillingness to see the partner again and perceptions that they were the source of infection also hindered notification of non-main partners. But, long-term non-main partners were sometimes notified to avoid the risk of re-infection.

Where participants had notified their partner, some had told their partner to *“go to the clinic”* as opposed to informing them about their STI diagnosis, which sometimes led to re-infection if their partner had then not done so:

*Participant: I said, “I’ve been to the clinic today and I’m not sure what it is yet, but they gave me tablets and I think you probably should go and have a check-up.” And that was it. But I think it’s because, I don’t know if she did go and got tested or not from the last time, and because I’ve been to Sweden and came back, and she is the only person that I have had intercourse with since I’ve been back, and I’ve got it again, so I think that it might be from the last time. And a couple of days before I got tested, I had had sex with her.*

***Interview 31, male age range 25-35 years, West London***

### Factors influencing concurrency

**Fig. 1:**
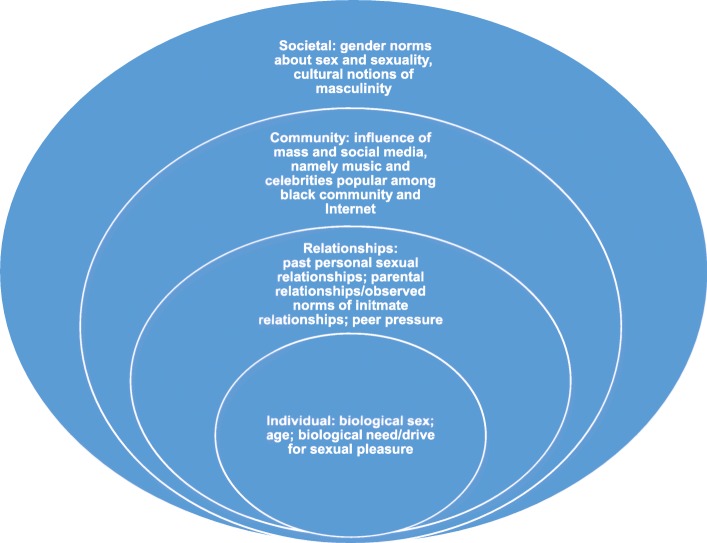
Ecosocial model of factors influencing concurrent partnerships among black Caribbeans in England

A complex interaction between the individual-level, relationship/interpersonal, community-level and societal factors (Fig. 1) influenced attitudes towards, and experiences of, concurrency among black Caribbeans. Focus group and interview data reflected that most factors influence both types of concurrency, although some factors facilitated specific type(s) of concurrency.

#### “Biological thing”

Lack of fulfilment of sexual needs by one sexual partner, need for *“variety”*, *“lust”* and it being a *“biological thing”* were often cited as drivers of concurrency, especially among men. This was further fuelled by perceived notions of *“West Indian”* mens’ masculinity:

*Interviewer: What makes you seek out others (partners) when you’ve got a regular partner?*

*Participant: Oh, it’s different, innit, that’s why, because obviously you meet an individual partner and if you have sex with them, that sex is different to the other person that you’re having sex with or whatever. I suppose you get bored of one thing. Like say I was wearing this coat all the time, all the time, and I thought to myself, you know what, I want to feel different or I want a different design, just get a different design. Like, I’m getting bored of this now. Or like you’re driving a car, you have a car for a few years and then you’re like, oh, I feel like a change, buy a new car, like that.*

***Interview 7, male age range 25-35 years, Birmingham***

*Male participant 1: You know, sometimes a man might have a woman for a long time and she’s not giving him what he wants, so he goes astray, he goes and looks for somebody outside. And then he’s still with you, he still loves you and he comes back with this disease!*

*Male participant 2: Yes, but it’s not only the men, women do it as well! I think women get fed up of men more quicker than a man getting fed up of a woman.*

***Focus group 4, mixed sex participants’ aged >45 years, South London***

Some female participants who reported having concurrent partnerships in the past due to lust for sex and variety had changed their behaviour after entering a committed relationship, especially after having children.

#### Past relationships

Both men and women who were/had been in main plus concurrent partnerships felt that meeting an ex-partner for shared childcare responsibilities was one of the most common contexts in which such partnerships occur.

*Participant: Just going back round there, chilling and then gathering our feelings again, innit, and then stay there and then next we just have sex. It became more casual kind of thing.*

***Interview 7, male age range 25-35 years, Birmingham***

Participants, especially women, felt that lack of self-esteem and self-confidence about finding another partner left them feeling lonely and vulnerable; like *“spoiled goods”*, which led them to continue to have sex with their ex-partner with whom they share a child. *“Unresolved feelings”* for an ex-partner (with whom a person does not necessarily share a child) was another reason cited for being in a main plus concurrent partnership. Some female participants, who were usually the non-main partner and aware of their partner’s main sex partner, cited *“being young”, “being in love”* as reasons for being in main plus concurrency.

Although not commonly reported, previous failed intimate relationships facilitated non-main concurrent partnerships:

*Participant: There was one time in my life and that was around the time that I had broken up with my daughter’s dad, that I had my casual partners. I was heartbroken so I was just like, something’s got to fill the void, which it didn’t and obviously that’s a life lesson. And it’s something that I’m not very particularly happy about, but you go through things and you learn about why it’s not good for you. At the end of the day, if you’re not with someone you might as well just wait and just work on you, rather than to be giving yourself and tiring out yourself for other people. Because sex is not just sex, it’s a connection.*

***Interview 11, female age range 25-35 years, East London***

#### Peer pressure

Both men and women talked about peer pressure to initiate sexual activity when young. Men also mentioned peer pressure to have multiple sex partners as a factor influencing their concurrent partnerships. This was influenced by cultural notions of black Caribbean men’s masculinity and media as described below. However, among young people and single people, non-main concurrency was common due to their pursuit for an appropriate long-term partner.

*Participant: I think it was peer pressure because if I was to turn back the hands of time, I don’t think I would have, but I was in a situation where it was just me and that other person, and they were like, “Oh, come on, come on!” So I think it was peer pressure, but I can’t put the blame on them, I played a part in it as well.*

***Interview 9, female age range 15-24 years, East London***

*Participant: It’s almost encouraged amongst young Black men by other young Black men, and older Black men, to be promiscuous, so I think that might be a reason.*

***Interview 3, male age range 25-35 years, Birmingham***

#### Parental/observed norms of intimate relationships

Some participants felt that some people engage in concurrency because it is a *“learnt behaviour”.* They *“think that’s the norm”* because they have seen their parent(s) or other family members do so and consciously or subconsciously they engage in similar relationships. Teenage pregnancies, single-parent households, and unmarried, non-cohabiting parents with both or either parent having other partner(s) were perceived to be common and enhance the practice of concurrency. Several participants had grown up in a single-parent family (usually women-led) with minimal to regular presence of a father and some with *“an absent father”,* i.e., he was never around when they were growing up. Participants felt that this influences young people’s attitudes towards sex, relationships, notions of family because they think that if their parent could do it, so could they*:*

*Participant: My mum’s partner lived with us. My dad was a fly-by-night type of guy; dad always lived by himself. He always had his own house and then whoever he was co-habiting with at the time, he’d be there for that period of time, but then if that went to pot, he would have somewhere to go back to.*

*Interviewer: And how did you feel about your parents’ relationships?*

*Participant: It was what it was, I didn’t know any different. So you’re learning from them without even knowing that you’re learning and they’re teaching you without them knowing that they’re teaching you.*

***Interview 1, female aged >35 years, Birmingham***

But not all participants who had experienced similar situations growing up felt the same:

*Participant: Growing up with my mum, my mum was there; she said that my dad was there, my biological dad was there, but I don’t remember, I’m too young to remember. I met him again at 15; I don’t really hold any grudges with him because, at the end of the day, people like to blame their papas, but for me that’s not really the case. Relationships grow, they work and they fail, and all you can do is thank them for trying their best, if they try their best, and try to understand the situation. Erm. I don’t know, I’ve just never been interested in being promiscuous like that, just having lots of different girlfriends at one time, it doesn’t appeal to me.*

***Interview 25, male aged >35 years, South London***

#### Mass media

Several participants felt that mass media plays a pivotal role in shaping, maintaining and facilitating cultural notions of masculinity, gendered norms of sex and sexuality which in turn influence attitudes towards concurrency. Some participants, especially younger ones, felt that glorification of partner concurrency via music that is popular among the black community, and via celebrity culture plays a role in normalising concurrency and even celebrating it. Some participants also felt that social media platforms such as Twitter are used, especially by men to applaud and encourage other men and sometimes to name and shame women perceived to have multiple partners. The changing norms and attitudes towards sex, facilitated by the ease of meeting sex partners online were also perceived to promote concurrency.

*Participant: I just think it’s the culture difference. So even, like, just down to the music that comes out of the Caribbean, it’s all about … well, not all, but a lot of it is about being sexually promiscuous! And I think even from a base level, things like that can subconsciously affect the minds of some people and they’ll think, OK, they’re doing it or celebrities are doing it, that’s what I can do as well, and it’s something I should be doing.*

***Interview 3, male age range 25-35 years, Birmingham***

*Participant 2: I think it’s (concurrent partnerships) always been there.*

*Participant 3****:****It’s always been there but I think now it’s almost glorified … … it’s like it’s not just social media, now it’s music as well. Like nearly every new kind of R&B or rap song that comes out, they are always talking about, OK, I’ve got my side bitch and I’ve this one and I’ve got that one (murmur of agreement) and, oh, my god, I’m so good, look at all my chains! And boys look at that and think I need to be that; that’s what girls want! But we don’t!*

***Focus group 1, young women aged 15-24 years, East London***

#### Notions of masculinity and gender

Most men and some women felt that the perceived notions of *“West Indian”* men as *“promiscuous”* facilitates concurrency. Some men also felt that women, particularly from non-black ethnicities prefer black men as sexual partners due to perceptions of their greater *“sexual prowess”*. Discussions about *“notches on bedposts”* among peers made some men feel that having multiple partners is *“something to be proud of”* and encouraged them to do so.

*Participant: The West Indian men that I know, a lot of them have got a very strong sexual drive and it pushes them to go that way, because sometimes their wife or girlfriend alone doesn’t sexually satisfy them. So they go somewhere else. Because I used to do that. It’s lust too. You see a nice girl walking down the street and you go, oh! Yes, I won’t lie about it. I used to have a lot of different girlfriends at one stage.*

***Interview 25, male aged >60 years, South London***

Concurrency was perceived to be a common practice among men; however it was felt that increasingly women too are engaging in concurrent partnerships. Participants, especially females, highlighted the *“double standards”* in attitudes towards men and women who have concurrent (and multiple) sexual partners. Unlike men, women were at a greater risk of stigma which was reflected in terms such as *“ho”* and *“slag”* being commonly used to describe women who engage in concurrency. Participants felt that concurrency is *“not acceptable, but men get away with it”* due to the *“traditional”* roles that are associated with being a woman or a man.

*Interviewer: And what about women having more than one partner at the same time?*

*Participant: For women, I don’t know if it’s a bit different or not, but the stigma of it these days is calling them slags, innit, like if a woman has more than one, it makes them look bad a bit; I don’t know why.*

*Interviewer: Who calls them that?*

*Participant: Men. Or women.*

*Interviewer: And where does that come from do you think?*

*Participant: Well, I think it’s died out now, because men get called dogs or whatever, but where do I think it’s come from? Don’t know, it’s come through traditional whatever. I couldn’t tell you really where it’s come from … Can’t think of a word, but that’s what women are perceived as, with more than one partner; for a man, it’s probably not looking that bad, but for a woman it is, for some reason.*

*Interviewer: And do you think that as well, generally what people are thinking?*

*Participant: Yeah, I kind of do, yeah, in a way. I don’t know. A little bit.*

***Interview 7, male aged 25-35 years, Birmingham***

*Female participant 3: We are called all names under the sun. Yeah, it’s not acceptable for a woman to carry herself like that, for some unknown reason; I have no idea why.*

*Female participant 2: I think people don’t think much of women who have multiple men.*

*Female participant 1: They’d look at her as a slut and say, “My god, how can you do that!?”*

*Male participant 4: Yeah, they’d put on her and call her a slut, all of these things, you know? All the loose words, you know? A woman get put down because of what she’s done, you know? (murmurs of agreement)*

***Focus group 3, mixed sex participants, age range 36-48 years, West London***

## Discussion

Our study conducted among black Caribbeans in England highlights that two key concurrency typologies exist among black Caribbeans, main plus and non-main, and their characteristics, especially awareness of partner concurrency and duration, influence sexual health choices and thus STI risk. Our results also highlight the range of emotional/psychological, interpersonal, sociocultural, and structural factors that can interact and shape the context in which concurrency occurs. In the following sections, we discuss the similarities and differences of our research findings in comparison to other research studies, and the implications of our study findings for clinical practice, research and policy.

### Implications for clinical practice

Similar to another study we found that one or both partners in a sexual relationship may have concurrent partners [26], which has implications for the spread of STIs if either partner is infected [9]. However, black Caribbean women commonly-described being in ‘closed-from-one-end’ main plus concurrent partnerships, implying that they are in a ‘passive’ concurrency, thus their STI risk is determined by their partner’s concurrent partnerships [27]. STI risk is likely to be especially heightened in the context of long-term and recurrent main plus concurrency due to an increased likelihood of condomless sex. Additionally often the lack of awareness of partner concurrency could hinder partner notification, which potentially explains the high rates of repeat STI diagnoses among black Caribbeans [28]. Conversely, irrespective of typology, perceived or actual awareness of partners’ concurrency facilitated condom use [14], STI testing, and partner notification. However, our data also suggest that condom use could change with changes in partnership status or increasing familiarity over time [29] and a mismatch of partnership expectations could also influence condom use and thus STI risk [27].

Because people may or may not be aware of their partner’s concurrency, which has been shown to be an independent predictor of STI risk [30, 31], regular STI testing and partner notification among black Caribbeans in concurrent partnerships should be promoted to prevent re-infections, and onward STI transmission. Collecting information from index patients in concurrent partnerships about the nature of the partnership(s) and their perceptions about their sex partners’ awareness of their concurrency could facilitate the process of offering partner notification by using methods that take account of concurrency type to facilitate case-finding. For example, using provider-led rather than patient-led partner notification if the index patient wants to remain anonymous in the event that their partner(s) is unaware of their concurrency.

Young participants were more likely than the older participants to report experiencing non-main concurrent partnerships. Younger people of all ethnicities generally are more likely to report other STI risk behaviours such as larger partner numbers than older people [32]. Additionally, young black Caribbeans are more likely than other ethnic groups to report early sexual debut [4]. This in part may explain the disproportionate STI burden experienced by young black Carribeans in England [2, 19]. Therefore promoting frequent STI testing among young people, especially young black Caribbeans is vital.

### Implications for research

Similar to studies conducted among African Americans in the USA, concurrency was often justified by participants in our study in the context of the need to satisfy sexual desires, and as a result of men’s biological needs [33, 34]. Moreover young people, especially men, were more likely to report peer pressure to have multiple sexual partners due to norms of black Caribbean men’s masculinity, perpetuated by social media; whereas psychosocial aspects usually influenced women’s decisions to be in concurrent partnerships. Perceptions of greater sexual prowess of black Caribbean men, particularly among women of non-black Caribbean ethnicity was mentioned by men in our interviews. These concur with the high prevalence of ethnic-mixing among black Caribbean men reported in a parallel quantitative study, although ethnic-mixing did not explain the high prevalence of STIs among these men compared to white British men [35]. The gendered double-standards towards concurrency reflected in the perceived greater tolerance of black Caribbean men - rather than women - having concurrent partnerships, tallies with greater prevalence of concurrency among black Caribbean men [4]. Modelling partnership patterns observed in one’s family/community [27, 34] appears to be one of the key factors influencing engaging in main plus concurrency among black Caribbeans. Sociological research is needed to understand the implications of familial structures on the sexual behaviour and thus sexual health of black Caribbeans. Our findings also highlight that future epidemiological studies should examine the prevalence of different types and characteristics of concurrency as they present varying levels of STI risk [13] and should assess the strength of their association with STI risk.

### Implications for policy

Current UK safer sex guidelines [36] recommend retesting for asymptomatic STIs to all individuals with a prior STI diagnosis including HIV. Given the complex range of factors that influence and sustain concurrent partnerships, enhancing STI risk, anyone in a concurrent partnership who is diagnosed with STI(s) should be retested. These guidelines also recommend screening for asymptomatic STIs at least annually (and in some cases as frequently as every 3 months) to all individuals at risk of acquisition or transmission of HIV. Although the risk of HIV is low among people of black Caribbean ethnicity, given the high STI burden among them, STI testing at least annually should be encouraged, especially among young black Caribbeans reporting concurrent partnerships.

Our data also suggest that gender-sensitive and age-specific, multi-faceted [37] interventions among black Caribbeans should be developed to address STI risks associated with different concurrency types, and psychosocial vulnerabilities that lead to maintenance of some concurrent partnerships. Given that STI diagnoses rates among black Caribbeans attending sexual health clinics in England are high [2], they provide a setting to offer interventions targeting black Caribbeans at STI risk. Interactive digital interventions are an effective means for promoting sexual health knowledge [38] and so could be used to offer STI risk-reduction interventions tailored to black Caribbeans reporting self or partner concurrency.

Our findings should be interpreted in the context of the following limitations. Unlike some USA studies, neither economic dependence nor the sex ratio [13, 39] were mentioned in our study as driving factors for concurrency, highlighting the significance of qualitative studies in understanding context-specific factors that influence behaviours. Nonetheless this could be an anomaly of our sampling strategy, and we recognise that the concurrency typologies described in our study may not be generalisable to the black Caribbean population across England. Nevertheless, we recruited our sample from areas in England with high concentrations of black Caribbeans and included men and women of different ages. Furthermore, by sampling participants from sexual health clinics and community-based organisations, we achieved a diverse group of black Caribbeans. We conducted all, except one, focus group discussions with mixed-sex groups, to facilitate an open discussion on social norms and practices related to concurrency among this ethnic group and to understand its gendered patterns. These were followed by one-to-one interviews, separately with men and women.

The gendered attitudes towards concurrency may have influenced reporting of concurrent partnerships. Moreover, the interviewers were mindful of the impact of stigma associated with concurrency and participants’ concerns about its discussion in the context of their ethnicity and STIs (especially as both interviewers were of non-black Caribbean ethnicity: one was white and one from another minority ethnic background). In an attempt to minimise the impact of their characteristics on participants’ reporting of sexual behaviours, prior to recruiting participants, interviewers explained the epidemiological evidence of high STI prevalence among black Caribbeans in England and the scientific importance of the information disclosed during interviews/discussions by participants for gaining a greater understanding of the factors influencing these STI trends. In this respect, it is worth noting the similarities in some of the themes identified by our study with previous studies on concurrency conducted in the UK which had ethnicity-matched interviewers [27] and from the USA where sex- and ethnicity-matched interviewers were used [34] suggesting that the information elicited from our study participants is reliable.

Finally, our interview data are not at a partnership-level that is, we did not interview all partners in a sexual relationship. We are therefore unable to confirm participants’ reports of their partners’ characteristics and behaviours. Perceptions of partners’ concurrency or lack of it, and its implications for sexual health choices therefore should be interpreted with caution.

## Conclusions

Overall, our findings suggest that concurrency type, its duration, and awareness influence sexual health choices, and thus STI risk among black Caribbeans. Collecting these data during clinic consultations could facilitate offering appropriate tailored interventions to black Caribbeans in concurrent partnerships. However, such interventions should factor in challenges posed by its role in enhancing sexual pleasure, and its social and structural determinants.

## Data Availability

The qualitative datasets generated and analysed during this study are not available as they run the risk of identifying individuals taking part. Only if additional external funds are available to anonymise this dataset could it be made available from the authors upon reasonable request, and with permission of University College London.

## Abbreviations

HPRU: Health Protection Research Unit
STI: Sexually transmitted infections
UCL: University College London
